# Benefits and risks of rapid initiation of antiretroviral therapy

**DOI:** 10.1097/QAD.0000000000001671

**Published:** 2017-12-01

**Authors:** Nathan Ford, Chantal Migone, Alexandra Calmy, Bernhard Kerschberger, Steve Kanters, Sabin Nsanzimana, Edward J. Mills, Graeme Meintjes, Marco Vitoria, Meg Doherty, Zara Shubber

**Affiliations:** a Department of HIV, World Health Organization, Geneva, Switzerland; b Centre for Infectious Disease Epidemiology and Research, University of Cape Town, Cape Town, South Africa; c HIV Unit, Division of Infectious Diseases, Geneva University Hospital, Geneva, Switzerland; d Médecins sans Frontières, Mbabane, Swaziland; e School of Population and Public Health, University of British Columbia, Vancouver, British Columbia, Canada; f HIV/AIDS and STIs Diseases Division, Rwanda Biomedical Centre, Kigali, Rwanda; g Basel Institute for Clinical Epidemiology and Biostatistics, University Hospital of Basel, Basel, Switzerland; h University of Rwanda, Kigali, Rwanda; i Department of Medicine, University of Cape Town Health Sciences Faculty, Cape Town, South Africa; j Department of Infectious Disease Epidemiology, Imperial College London, London, UK.

**Keywords:** antiretroviral therapy, rapid initiation, same day start

## Abstract

**Background:**

Recent attention has focused on the question of how quickly antiretroviral therapy (ART) should be started once HIV diagnosis is confirmed. We assessed whether rapid ART initiation improves patient outcomes.

**Methods:**

We searched five databases from inception up to August 2017. Rapid ART initiation was defined as initiation within 14 days of HIV diagnosis. Data were pooled using random effects meta-analysis.

**Results:**

Across the randomized trials, ART start on the same day increased viral suppression at 12 months [three trials: relative risk (RR) 1.17, 95% confidence interval (CI) 1.07–1.27], retention in care at 12 months (RR 1.11, 95% CI 0.99–1.26), and the likelihood of starting ART within 90 days (four trials: RR 1.35, 95% CI 1.13–1.62) and 12 months after eligibility was established (three trials: RR 1.17, 95% CI 1.07–1.27). There was a nonsignificant trend toward reduced mortality (three trials: RR 0.53, 95% CI 0.24–1.08), as well as reduced loss to follow-up at 12 months (2 trials: RR 0.66, 95% CI 0.42–1.04). In the observational studies, offering accelerated ART initiation resulted in a greater likelihood of having started ART within 3 months (two studies: RR 1.53, 95% CI 1.11–2.10). There was a trend toward an increased risk of being lost to follow-up at 6 months (three studies: RR 1.85, 95% CI 0.96–3.55).

**Conclusion:**

Accelerated ART initiation can lead to improved clinical outcomes and is likely to be of particular benefit in those settings where extensive patient preparation prior to starting ART results in long delays. These findings informed a WHO recommendation supporting accelerated ART initiation, including same day ART start.

## Introduction

The question of when to start antiretroviral therapy (ART) in people living with HIV (PLHIV) has been a major focus of research and policy over the last two decades. Following the results of two large randomized trials demonstrating a clinical benefit to starting ART at any CD4^+^ cell count [1,2], there has been a rapid shift in global guidelines toward adopting a policy of treating all PLHIV as soon as an HIV diagnosis is confirmed [3].

More recent attention has focused on the question of how quickly ART should be started once HIV diagnosis is confirmed. [4] In the early years of the response to HIV, limited resources and concerns about suboptimal treatment adherence led to a cautious approach whereby PLHIV underwent multiple counselling sessions that could last several weeks or months prior to start of ART [5]. During this period prior to ART initiation, substantial attrition was observed [6], leading research to focus on whether accelerated approaches to ART initiation, including initiation on the same day that HIV is diagnosed or eligibility is determined, could reduce loss to care prior to start of ART and improve clinical outcomes. The question of how quickly ART should be started is all the more relevant in the ‘treat all’ era, and several national guidelines in high income and resource-limited settings have recently been revised to recommend rapid ART initiation in certain situations, including pregnancy [7], patients with advanced HIV disease [8], and for people with acute HIV infection [9].

The results of several recent randomized trials have indicated that accelerated ART initiation, including same day start, can improve patient and programme outcomes, in particular by reducing loss to care in the period prior to ART initiation [10,11]. However, there is some evidence from pregnant women receiving care in the context of routine programmes that rapid initiation can lead to increased loss to follow-up post-ART initiation likely because of insufficient time to accept and disclose HIV status [12].

The systematic review, aiming to address the knowledge gap around programme implications of rapid ART initiation, was conducted to inform the development of WHO guidance on accelerated ART initiation.

## Methods

### Search strategy and selection criteria

The systematic review was conducted according to the Preferred Reporting Items for Systematic Reviews and Meta-analysis (PRISMA) guidelines [13] and followed a study protocol (available from the corresponding author).

Randomized and quasi-randomized controlled trials, comparative and noncomparative observational studies, and qualitative studies reporting outcomes of accelerated ART initiation (defined as offering three or more antiretroviral drugs as part of combination ART ≤14 days post eligibility ascertainment) were included. No language or geographical restrictions were applied. Reasons for exclusions included individuals receiving antiretroviral drugs for reasons other than treatment of HIV infection (e.g. ART for postexposure prophylaxis and preexposure prophylaxis), and studies including patients with coinfections for whom immediate ART is not advised for clinical reasons (e.g. cryptotoccal meningitis). The minimum sample size for inclusion was defined as at least 20 patients.

Using a broad search strategy combining terms for HIV infection, ART, and treatment initiation, two investigators (N.F., Z.S.), working independently and in duplicate, screened titles and abstracts from MEDLINE via PubMed, Embase, the Cochrane library, LILACS, and Web of Science from inception to 1 August 2017. Abstracts from the International AIDS Society conferences (from 2012 to 2016) and the Conferences on Retroviruses and Opportunistic Infections (from 2012 to 2017) were also screened to identify studies that have been recently completed but not yet published in full. Ongoing trials were sought via ClinicalTrials.gov. Database searches were supplemented by screening bibliographies of review articles and all included full-text articles. The same investigators scanned all abstracts and full-text articles and achieved consensus on final study inclusions.

### Data extraction

The same two reviewers working independently (N.F., Z.S.) extracted data following a predefined protocol and using a standardized and piloted extraction form. Study characteristics included design, year, population, location, ART eligibility, and interventions. Extracted clinical outcomes included the proportion of PLHIV starting ART as well as data on: loss to follow-up, mortality, retention in care, and virological suppression. Information about patient characteristics (age, pregnancy status, and having acute vs. chronic infection) and indicators of study quality (that were used to inform an overall assessment of the certainty of the evidence using the Grading of Recommendations Assessment Development and Evaluation (GRADE) approach [14]) were also extracted.

### Statistical analysis

For each of the randomized trials and comparative observational studies relative risks (RR) and corresponding 95% confidence intervals (CIs) were calculated and data were pooled by study design using random effects meta-analysis [15]. The pooled proportion of individuals who started ART on the same day that HIV was diagnosed was determined for the observational studies and was calculated as a measure of acceptability of the intervention in programme settings. Randomized trials and observational studies were analysed separately, with adjustment made for clustering in the large cluster trial [16]. We analysed all data with Stata version 13.0 (Stata Corp., College Station, Texas, USA).

## Results

### Characteristics of included studies

**Fig. 1:**
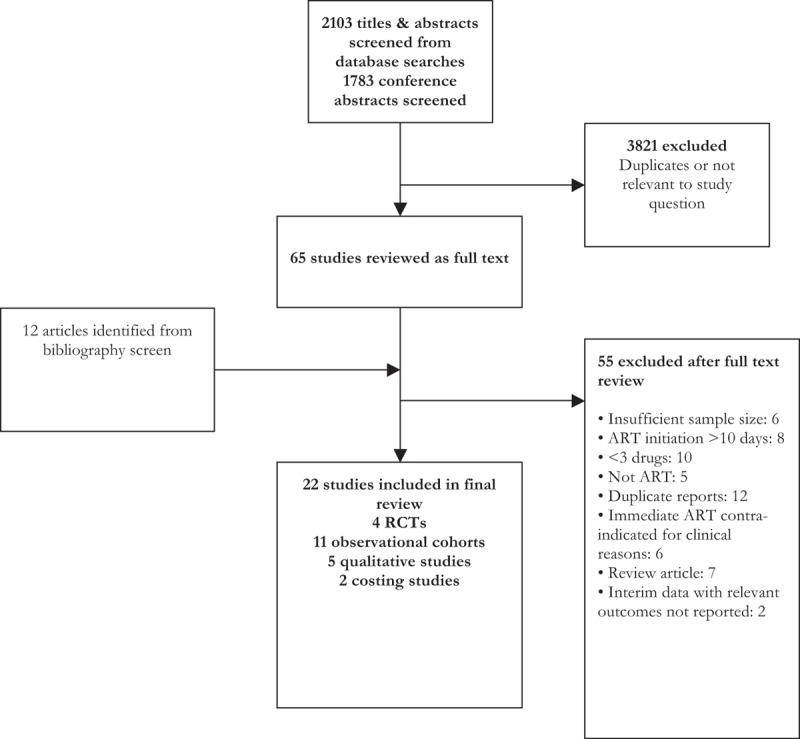
Study selection process.

ART, antiretroviral therapy; arct, randomized controlled trial.

From an initial screen of 3886 titles, 22 studies were included in this review, comprising two individual randomized controlled trials (Haiti and South Africa) [11,17], two cluster randomized trials (Uganda and Lesotho) [10,18], 11 observational studies carried out across eight countries (China, Ethiopia, Malawi, South Africa, Swaziland, Thailand, the United Kingdom, and the United States) [19–29], and five qualitative studies (Fig. 1) [12,30–33]. Information on cost was reported for one of the trials [34] and two additional observational studies [29,35]. Two additional ongoing randomized controlled trials provided some initial results but data were not available for the outcomes of interest and these studies are not included in this review [36,37]. Characteristics of included studies are provided in Supplementary Table 1.

Three of the trials were carried out among nonpregnant, HIV-infected adults [11,17,18]; in the fourth trial around a quarter of female participants were pregnant [10]. Cointerventions were provided as part of the intervention arm in two of the trials, and included point-of-care CD4^+^ cell testing [10,11], revised counselling to support readiness [10,11], and facility feedback on ART initiation rates [10]. Three of the trials offered ART initiation on the same day that HIV was diagnosed [17,18], one offered ART initiation within 2 weeks of having established eligibility for treatment (with the majority starting on the same day) [10] while the forth trial offered ART initiation on the same day as the first HIV-related clinic visit following a positive HIV diagnosis [11]. The observational studies provided additional data on pregnant women [19,20,23–25] and people diagnosed with an acute HIV infection [21,22,26,27]. Four of the observational studies were noncomparative studies [19,21,27,28]; one study compared data pre and postintervention [29], and the remainder provided contemporaneous comparisons for patients starting ART at different time points within the same cohort [20,22–26].

The quality of the evidence from the four randomized trials was rated as high for the majority of outcomes. Overall, risk of bias was judged to be low and the certainty of the evidence was judged to be high-to-moderate. For the comparative observational studies the certainty of the evidence overall was rated as low because of imprecision and risk of bias associated with the use of a retrospective study design (three studies) and because some outcomes were only reported for a subset of PLHIV. The risk of bias assessment is summarized in Supplementary Table 2.

### Outcomes from randomized trials

**Fig. 2:**
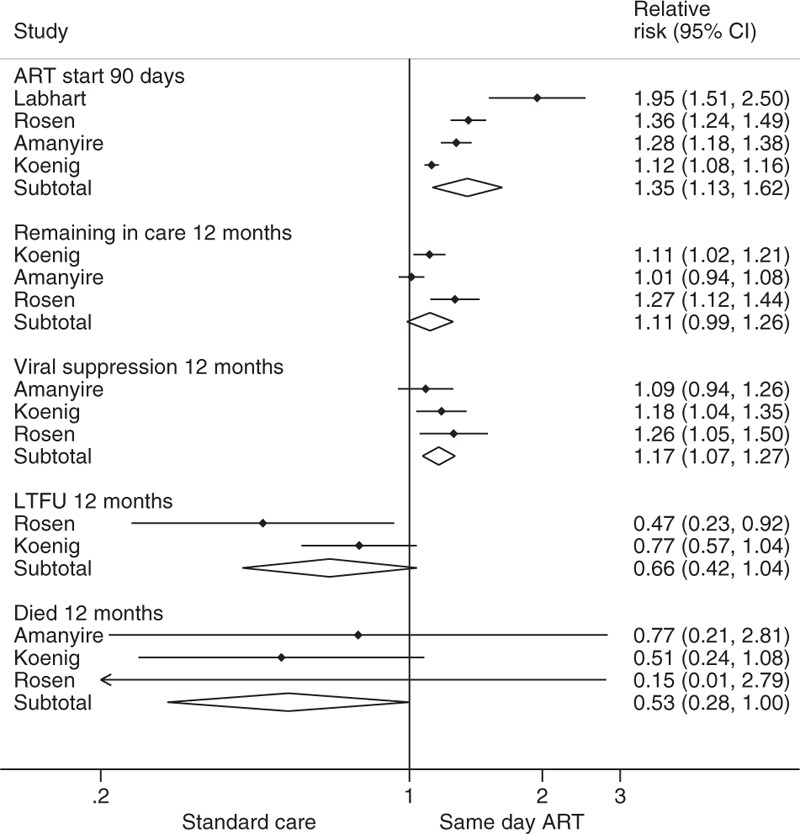
Outcomes from randomized trials comparing same day ART start vs standard of care.

ART, antiretroviral therapy; CI, confidence interval.

All four randomized controlled trials provided data on the effect of initiating ART on the same day that eligibility was established/first clinic visit on clinical outcomes. There was high-to-moderate quality evidence of benefit with respect to all clinical outcomes assessed, including evidence that ART start on the same day increased viral suppression at 12 months (three trials: RR 1.17, 95% CI 1.07–1.27) and retention in care at 12 months (RR 1.11, 95% CI 0.99–1.26). ART start on the same day also increased the likelihood of starting ART within 90 days (four trials: RR 1.35, 95% CI 1.13–1.62) and 12 months after eligibility was established (three trials: RR 1.17, 95% CI 1.07–1.27). There was a nonsignificant trend toward reduced mortality (three trials: RR 0.53, 95% CI 0.24–1.08). Loss to follow-up at 12 months (two trials: RR 0.66, 95% CI 0.42–1.04) was also reduced, although this reduction was not statistically significant (Fig. 2).

### Outcomes from observational cohorts

Eight observational studies provided data on outcomes comparing accelerated ART initiation (same day start [20,22–26] or ART start within 5 days [29]) with standard of care. Offering accelerated ART initiation resulted in a greater likelihood of having started ART within 3 months (two studies: RR 1.53, 95% CI 1.11–2.10; low-quality evidence). However, there was no evidence that offering accelerated ART start resulted in a greater likelihood of remaining in care (two cohorts: RR 0.97, 95% CI 0.79–1.18; low-quality evidence). One study reported an increased risk of being lost to follow-up at 3 months (RR 1.97, 95% CI 1.21–3.20), while two other studies reported a trend toward an increased risk of being lost to follow-up at 6 months (RR for the three studies: 1.85, 95% CI 0.96–3.55; very low-quality evidence). There was some evidence of a tendency toward a decreased likelihood of being virally suppressed at 6 months (one cohort: RR 0.93, 95% CI 0.87–1.00; very low-quality evidence).

The remaining four observational studies [19,21,27,28] did not provide within-study comparisons and were not included in the meta-analysis. A study from South Africa reported that 75.8% of pregnant women were virally suppressed at the time of delivery following same day ART initiation [19]. In contrast, studies among acutely infected MSM in Thailand [27], the United Kingdom [21], and the United States [22] reported higher levels of viral suppression at 6 months (>90%) following same day ART initiation. Finally, a study among adults in South Africa reported high rates of viral suppression (94%) among adults living with HIV offered accelerated ART initiation (average within 5 days of diagnosis) [28].

### Acceptability

Uptake of the offer of ART initiation on the same day that eligibility was determined was reported by five studies [20–22,27,33]. For individuals with acute HIV infection (mainly MSM), uptake ranged from 28.4% (95% CI 15.2–43.8%) in the United States [22] to 97.7% (95% CI 95.8–100) in Thailand [27]. For pregnant women, uptake ranged from 36.1% (31.1–41.2) in Malawi [20] to 90.5% (95% CI 84.9–94.9) in South Africa [19].

Barriers and concerns reported by PLHIV that could be specifically related to same day ART start were principally described in African studies, as well as a Thai study. They included insufficient time to process information for pregnant women [33], limited time to disclose HIV status that could potentially result in stigma and conflict, including domestic violence [12], a desire among pregnant women to seek partner approval prior to starting ART [30], uncertainty about the HIV test result, and the need for confirmatory testing [30]. Other reported barriers, including concerns about side-effects and the challenge of adhering to lifelong therapy and pill burden [19,31], were not considered to be specifically related to same day ART start. Provider reported barriers, reported by one of the trials, included the need for exclusion of tuberculosis (TB), having a WHO clinical stage 3 or 4 condition or the need for TB treatment (25/48, 52%), insufficient time to complete all steps on the same day (13%), and individual preference (10%) [11]. Reported enablers to same day ART start included a desire to prevent onward transmission among both pregnant women [33] and MSM [31], supportive counselling [12,30], and a perception that starting ART as soon as possible would reduce the risk of stigma [31].

### Cost and cost-effectiveness

Three studies reported on the cost and cost-effectiveness of accelerated ART initiation [29,34,35]. A study from South Africa that introduced a package of interventions to expedite ART initiation in pregnant women found that the package was very cost-effective compared to standard services (US$1160 per quality-adjusted life year saved) [35]. A second study, also from South Africa, found that same day treatment initiation using point-of-care tests is more effective and more expensive than standard initiation (incremental cost-effectiveness ratio US$780 per additional patient suppressed by 10 months); the increased costs were mainly driven by the use of point-of-care CD4^+^ cell count as part of the rapid ART package [34]. Finally, a study from China reported that the unit cost for an additional patient receiving ART under the simplified testing and treatment approach was US$83.80, declining to US$9.69 in the second year; this represented an effective and sustainable intervention, according to the study investigators [29]. The trial conducted in Haiti reported that cost and cost-effectiveness were assessed as secondary outcomes; however, no cost information was provided in the trial publication [17].

## Discussion

We found that accelerated ART initiation, including starting ART on the same day as HIV diagnosis, can lead to improved clinical outcomes by increasing the number of people starting and remaining on ART. Rapid ART start may be especially important for people with very low CD4^+^ cell counts, for whom the risk of death is high.

Not all patients may be ready to start treatment on the same day that diagnosis is confirmed. Nevertheless, people with no contraindication to early ART initiation should be fully informed of the benefits of early ART and offered rapid ART initiation, including the option of same day initiation.

In 2016, WHO issued guidelines recommending that countries adopt a policy of initiating ART as soon as possible after a positive HIV diagnosis is confirmed. At that time, there were limited data available to support the formulation of a recommendation on accelerated ART initiation, and these guidelines were limited to providing a good practice statement that efforts should be made to reduce the time between HIV diagnosis and ART initiation based on an assessment of a person's readiness [38]. In the absence of a formal recommendation, national guidelines have adopted different approaches to accelerated ART initiation. Kenya's guidelines recommend considering ART initiation on the same day as enrolment into HIV care for all patients [39]. Malawi [7] recommends offering ART on the same day of diagnosis to pregnant women. In Nigeria it is recommended that treatment should be initiated with an ‘increased sense of urgency’ [40].

As a result of the findings of this review, WHO now recommends that ART is initiated within 7 days following a confirmed HIV diagnosis and clinical assessment, and that ART initiation on the same day as HIV is diagnosed should be offered to those patients who are ready to start [41].

Accelerated ART initiation is likely to be of particular benefit in those settings where extensive patient preparation prior to starting ART results in long delays, during which patients may be lost to care [28]. Individuals most likely to benefit from this approach include those with advanced HIV disease (to reduce the high risk of mortality in this group), pregnant women, and those with acute HIV infection (to decrease viral load and reduce the risk of virus transmission).

There are some important concerns with respect to rapid ART initiation, notably with respect to the risk of immune reconstitution inflammatory syndrome; patients should undergo history and clinical examination to look for significant opportunistic infections (e.g. signs and symptoms of TB and those suggestive of meningitis) supported by diagnostic testing [cryptococcal antigen test, Xpert, and lipo-arabinomannan agent (TB-LAM)] where indicated, prior to being offered rapid ART, and ART initiation should be deferred when clinical symptoms suggest TB or cryptococcal meningitis to avoid paradoxical worsening of the existing infection that can be life threatening [42].

There is some evidence from observational studies that starting ART on the same day as HIV diagnosis may increase the risk of loss to follow-up, particularly among pregnant women, indicating the need for adapted counselling and continued research. In particular, implementation science research and programme monitoring should be supported to learn lessons from programmes that are implementing this approach at scale to inform future policy and practice. Finally, patients should not be coerced to start immediately, and should be supported in making an informed choice regarding when to start ART. These concerns are highlighted in a good practice statement that is included as part of the WHO recommendation on rapid ART initiation [41].

We used a broad search strategy and inclusion criteria that allowed us to identify evidence from a range of settings and populations. Evidence from observational studies are generally regarded as being of lower quality compared with randomized trials [43] and some systematic reviewers advocate excluding observational data when data from randomized trials are available. For this issue, however, we considered it important to assess outcomes of ‘real-world’ effectiveness alongside those reported by trials, which in general apply strict inclusion criteria and provide more support to patients than is normal in real-world settings. Most studies included in this review had a relatively short follow-up period, reporting outcomes up to 1 year. Nevertheless, it can be expected that most risks and benefits associated with accelerated ART initiation accrue in the initial months after ART is started. There is some evidence that outcomes in the initial months following ART initiation are predictive of outcomes over the longer term [44,45], underscoring the importance of ensuring patient preparation and counselling is provided in the first crucial months following ART initiation. Finally, although our search strategy was not limited in geographical scope, most of the evidence identified by this review, including all of the randomized trials, were conducted in low-to-middle-income countries with a high burden of HIV. As such, it is possible that the findings of this review have limited applicability beyond these settings.

In conclusion, the findings of this review suggest that accelerated ART initiation, including starting the same day as an HIV-positive diagnosis is confirmed, generally leads to improved outcomes. Careful attention is required to ensure that patients make an informed choice when offered ART.

## Acknowledgements

We thank Elui Batya, Tracy Glass, Martin Hoenigl, Mark Hull, Niklaus Labhart, and Gary Whitlock for providing additional data and clarifications on their studies. G.M. is supported by the Wellcome Trust (098316) and the South African Research Chairs Initiative of the Department of Science and Technology and National Research Foundation (NRF) of South Africa (Grant No 64787).

N.F. and A.C. conceived the review. N.F., Z.S., and C.M. undertook the literature review and data extraction. Statistical analyses were carried out by N.F., with inputs from S.K. and E.J.M. All authors provided critical input to the interpretation of the data, contributed to the drafting of the manuscript, and approved the final version.

### Conflicts of interest

There are no conflicts of interest.

## Supplementary Material

Supplemental Digital Content
