# Supplementary material for: Benefits and risks of rapid initiation of antiretroviral therapy
Source: AIDS. 2017 Dec 1;32(1):17–23. doi: 10.1097/QAD.0000000000001671 (PMC5732637; doi:10.1097/QAD.0000000000001671)
Supplement: Supplemental Digital Content [file aids-32-17-s001.docx]

| **Section/topic** | **#** | **Checklist item** | **Reported on page #** |
| --- | --- | --- | --- |
| **TITLE** | | |  |
| Title | 1 | Identify the report as a systematic review, meta-analysis, or both. | 1 |
| **ABSTRACT** | | |  |
| Structured summary | 2 | Provide a structured summary including, as applicable: background; objectives; data sources; study eligibility criteria, participants, and interventions; study appraisal and synthesis methods; results; limitations; conclusions and implications of key findings; systematic review registration number. | 2 |
| **INTRODUCTION** | | |  |
| Rationale | 3 | Describe the rationale for the review in the context of what is already known. | 3 |
| Objectives | 4 | Provide an explicit statement of questions being addressed with reference to participants, interventions, comparisons, outcomes, and study design (PICOS). | 4 |
| **METHODS** | | |  |
| Protocol and registration | 5 | Indicate if a review protocol exists, if and where it can be accessed (e.g., Web address), and, if available, provide registration information including registration number. | 4 |
| Eligibility criteria | 6 | Specify study characteristics (e.g., PICOS, length of follow-up) and report characteristics (e.g., years considered, language, publication status) used as criteria for eligibility, giving rationale. | 3-4 |
| Information sources | 7 | Describe all information sources (e.g., databases with dates of coverage, contact with study authors to identify additional studies) in the search and date last searched. | 4 |
| Search | 8 | Present full electronic search strategy for at least one database, including any limits used, such that it could be repeated. | Protocol |
| Study selection | 9 | State the process for selecting studies (i.e., screening, eligibility, included in systematic review, and, if applicable, included in the meta-analysis). | 4 |
| Data collection process | 10 | Describe method of data extraction from reports (e.g., piloted forms, independently, in duplicate) and any processes for obtaining and confirming data from investigators. | 4 |
| Data items | 11 | List and define all variables for which data were sought (e.g., PICOS, funding sources) and any assumptions and simplifications made. | 4 |
| Risk of bias in individual studies | 12 | Describe methods used for assessing risk of bias of individual studies (including specification of whether this was done at the study or outcome level), and how this information is to be used in any data synthesis. | 5 |
| Summary measures | 13 | State the principal summary measures (e.g., risk ratio, difference in means). | 5 |
| Synthesis of results | 14 | Describe the methods of handling data and combining results of studies, if done, including measures of consistency (e.g., I^2^) for each meta-analysis. | 5 |

Page 1 of 2

| **Section/topic** | **#** | **Checklist item** | **Reported on page #** |
| --- | --- | --- | --- |
| Risk of bias across studies | 15 | Specify any assessment of risk of bias that may affect the cumulative evidence (e.g., publication bias, selective reporting within studies). | 5 |
| Additional analyses | 16 | Describe methods of additional analyses (e.g., sensitivity or subgroup analyses, meta-regression), if done, indicating which were pre-specified. | 5 |
| **RESULTS** | | |  |
| Study selection | 17 | Give numbers of studies screened, assessed for eligibility, and included in the review, with reasons for exclusions at each stage, ideally with a flow diagram. ` | 5, Figure 1 |
| Study characteristics | 18 | For each study, present characteristics for which data were extracted (e.g., study size, PICOS, follow-up period) and provide the citations. | 5 Supplementar Appendix |
| Risk of bias within studies | 19 | Present data on risk of bias of each study and, if available, any outcome level assessment (see item 12). | Supplementary Appendix |
| Results of individual studies | 20 | For all outcomes considered (benefits or harms), present, for each study: (a) simple summary data for each intervention group (b) effect estimates and confidence intervals, ideally with a forest plot. | 5, Figure 2 |
| Synthesis of results | 21 | Present results of each meta-analysis done, including confidence intervals and measures of consistency. | 5, Figure 2 |
| Risk of bias across studies | 22 | Present results of any assessment of risk of bias across studies (see Item 15). | Supplementary Appendix |
| Additional analysis | 23 | Give results of additional analyses, if done (e.g., sensitivity or subgroup analyses, meta-regression [see Item 16]). | 5-9 |
| **DISCUSSION** | | |  |
| Summary of evidence | 24 | Summarize the main findings including the strength of evidence for each main outcome; consider their relevance to key groups (e.g., healthcare providers, users, and policy makers). | 8-10 |
| Limitations | 25 | Discuss limitations at study and outcome level (e.g., risk of bias), and at review-level (e.g., incomplete retrieval of identified research, reporting bias). | 8-10 |
| Conclusions | 26 | Provide a general interpretation of the results in the context of other evidence, and implications for future research. | 8-10 |
| **FUNDING** | | |  |
| Funding | 27 | Describe sources of funding for the systematic review and other support (e.g., supply of data); role of funders for the systematic review. | 2 |

*From:*  Moher D, Liberati A, Tetzlaff J, Altman DG, The PRISMA Group (2009). Preferred Reporting Items for Systematic Reviews and Meta-Analyses: The PRISMA Statement. PLoS Med 6(6): e1000097. doi:10.1371/journal.pmed1000097

For more information, visit: **www.prisma-statement.org**.

**Table 1a Study characteristics: Randomized trials**

| Study | Country | Sample | Design | Timeframe | Population | Median CD4 at ART initiation,  mm^3^ (IQR) | ART eligibility | Exclusions | Intervention | Co-interventions | Comparator |
| --- | --- | --- | --- | --- | --- | --- | --- | --- | --- | --- | --- |
| Amanyire^1^ | Uganda | 12024 | Cluster RCT (step-wedged) | 2013-2015 | Adults (>18 years) | 310 (179–424) | CD4 <350, WHO Stage III/IV, pregnancy, later included key populations + sdc + CD4 <500 | None | ART initiation within 14 days of eligibility determination  (Same Day ART start was reported as a secondary endpoint) | Training and coaching, PoC CD4, revised counselling approach and facility feedback | SoC (variable but usual practice included 3 pre treatment counselling sessions,  requirement for treatment supporter, + overnight CD4  cell count processing);  ART at 28 days (mean) |
| Koening^2^ | Haiti | 703 | RCT | 2014-2016 | Adults | 248 (148-345) | CD4<500 | Pregnancy,  TB or pneumonia; failure to demonstrate “pre-preparedness” | Same Day ART (same day as HIV diagnosis) | SoC (with the exception of ART start timing)  Chest x-ray | SoC= Counselling/ social worker visits^1^  and transportation subsidy  ART at day 21 |
| Rosen^3^ | South Africa | 463 (377 eligible for ART) | RCT | 2013-2014 | Adults (>18 years) | Median: <200  Rapid arm: 224 (128–327)  SoC arm: 195 (103–322) | CD4 <350 or WHO Stage III/IV | Pregnancy | Same Day ART (first HIV-related clinical visit) | PoC CD4, rapid TB test and PoC baseline blood tests  Group and individual counseling as per SoC also provided but condensed version provided all on same day | ART at visit 6 (2-4 weeks post visit 1; mean 22 days, Mean 22 days)^2^  Group and individual counseling |
| Labhardt^4^ | Lesotho | 276 | Cluster RCT | -2017 | Adults (>18 years) | 380 (249-530) | Treat All | Pregnant or breast-feeding, already enrolled in care for another chronic disease, clinical WHO stage 4 or active tuberculosis, or a positive cryptococcal antigen test | Same Day ART (same day as HIV diagnosis) | Home-based HIV testing | Post-test counselling and referral to nearest HIV-clinic |

^1^ Day 1, 3, 10, and 17 and for missed visits for intervention and Day 7, 14, and 21 and for missed visits for control group.

^3^ Fast-track (1 week) initiation for patients who are very ill or have very low CD4 cell count

ART= antiretroviral therapy; Hb= haemoglobin; IQR= interquartile range; NR= not reported; PoC= point-of-care; RCT= randomized controlled trial; sdc= sero-discordant couples; SoC= standard of care; TB= tuberculosis

**Table 1b: Study characteristics: observational studies**

| Study | Country | Sample | Design | Timeframe | Population | Median CD4 at ART initiation | ART eligibility | Intervention | Comparator |
| --- | --- | --- | --- | --- | --- | --- | --- | --- | --- |
| Comparative studies | | | | | | | | | |
| Chan^5^ | Malawi | 344 | Retrospective cohort | 2011-2012 | Pregnant women | NR | PMTCTB+ | ART integrated into ANC, including same day start | Integration of HTC with ANC only and initiation at visit to ART care |
| Hoenigl^6^ | USA | 22 | Retrospective cohort | 2010-2015 | Adults | 466 cells/mm^3^ | Treat All | Same day ART | Later ART start (within 4 weeks but not same day) |
| Kerschberger^7^ | Swaziland | 1243 | Prospective cohort | 2014-2016 | Pregnant and non-pregnant adults (≥16 years) | 313 (158-474) | Treat all versus CD4< 350 & CD4<500 (SoC); PMTCTB+ for pregnant women | Same day ART for pregnant women; Rapid ART for non-pregnant adults: ART initiation on the same day as HIV diagnosis and HIV care enrolment. | Deferred ART whereby ART is initiated after HIV diagnosis and care enrolment (median days between diagnosis and ART= 14 (7-27) |
| Langwenya^8^ | South Africa | 618 | Prospective cohort | 2013-2014 | Pregnant women | NR | April-July 2013: CD4<350; July 2013 – June 2014: PMTCTB+ | Immediate ART (Same day as first ANC visit) | SoC= ART usually 1-2 weeks after first ANC visit |
| Mitiku^9^ | Ethiopia | 346 | Retrospective cohort | 2013-2015 | Pregnant and breastfeeding women | 460 (277-638) | PMTCTB+ | ART integrated into ANC, including same day start. Those with Stage III/IV disease are referred to ART clinic. | Later than same day |
| Pilcher^10^ | USA | 86 | Retrospective cohort | 2013-2015 | Adults | Rapid: 474 (3-1391)  SoC: 417 (11 -1194) | Treat All if acute/ recent infection, CD4<200, active OI, & sdc | Same Day ART | ART prescribed a median of 22 days after referral to clinic |
| Wu^11^ | China | 1034 | Pre- and post-intervention | Pre-intervention: 2010-2011  Post-intervention: 2012-2014 | Adults | Pre-intervention: 2010= 243 (75-384); 2011= 219 (77-403); Post-intervention: 2012-2013= 220 (69-379)  2013-2014= 178 (53-330) | Pre-intervention CD4 <350; Post-intervention Treat all (but outcomes reported only for those with CD4<350) | Post intervention median time to ART: 5 days | Median time to ART 43-53 days |
| Non-comparative studies | | | | | | | | | |
| Black^12^ | South Africa | 134 | Prospective cohort | 2011-2012 | Pregnant women | 241-244 | CD4 <350  Stage III/IV | Rapid initiation at pregnancy, including integrated ART/ANC services allowing same day ART in some cases, single counseling session & PoC CD4 | None |
| Girometti^13^ | United Kingdom | 113 | Retrospective cohort | 2014-2015 | Acutely infected adults (MSM) | 483 (351-701) | Treat All | Initiation of ART offered at first clinic visit (usually 2 weeks after HIV diagnosis) | None |
| De Souza^14^ | Thailand | 112 | Prospective cohort | 2009-2014 | Acutely infected adults (96% men; 92% MSM/bisexual) | 353 cells/mm^3^ | Treat all | Initiation of ART offered at first clinic visit | None |
| Wilkinson^15^ | South Africa | 449 | Prospective cohort | 2012-2014 | Adults | 242 (147-308) | CD4 <350  Stage III/IV | Reduced pre-ART counseling sessions from 3 weeks to same day (unless contra-indicated, e.g. TB coinfection). ART initiation at second visit | None |

ANC= antenatal care; ART= antiretroviral therapy; HTC= HIV testing and counseling; MSM= men who have sex with men; NR= not reported; OI= opportunistic infection; PMTCTB+= Prevention of Mother-To-Child HIV Transmission Option B+; PoC= point-of-care; sdc= sero-discordant couples; TB= tuberculosis

**Table 1c: Qualitative studies**

| **Study** | **Focus** | **Design** | **Year** | **Population** | **Age** | **Country** | **Rural/ urban** | **ART eligibility** | **Intervention** | **Comments** |
| --- | --- | --- | --- | --- | --- | --- | --- | --- | --- | --- |
| Black et al, 2014 | Acceptability and challenges of the rapid initiation of ART among pregnant women living with HIV | 7 key informant interviews and semi-structured interviews with participants | 2011 | HIV positive pregnant and postpartum women | Mean 29 years (range: 23–36) | South Africa | Urban | PMTCTB+ | Rapid initiation at pregnancy, including integrated ART/ANC services allowing same day ART in some cases, single counseling session & PoC CD4 | Same intervention as reported by Black et al, 2013, and Myer et al, 2012 |
| Helova et al, 2016 | Challenges to the provision  of PMTCTB+  at the health facility level | 40 individual gender-matched one-on-one in-depth interviews with HIV-positive pregnant or postpartum women (n=20) and their male partners (n=20) and 4 FGD with 30 healthcare providers | 2014 | HIV positive pregnant and postpartum women | Pregnant women 24.7; male partners 33.5; healthcare workers 32.2 | Kenya | Rural | PMTCTB+ | Same day ART |  |
| Katirayi et al, 2016 | Barriers and facilitators that affect a woman’s decision to initiate and to adhere to PMTCTB+ | 39 in-depth interviews (pregnant and postpartum women) and 16 FGD (4 healthcare workers, 8 pregnant and postpartum women) | 2013 | HIV positive pregnant and postpartum women | Mean age 27.2-29.5 | Malawi | Rural, urban and periurban | PMTCTB+ | Same day ART |  |
| Maek-a-nantawat et al, 2014 | Attitudes toward and interest in regular HIV testing and immediate ART | Self-administered questionnaire before and after HIV testing | 2011-2012 | 434 MSM (69 HIV positive) | Median 26 (IQR 22–31) | Thailand | Urban | Treat all | Annual HIV testing and immediate ART | Unclear if immediate ART necessarily refers to same day |
| Nakanwagi et al, 2016 | Identify the facilitators and barriers to linkage to HIV care among HIV positive FSWs | 28 in-depth interviews with FSW and key informant interviews (5 project staff and 11 peer educators) | 2012-2013 | FSW | 85% 20–30 years; 15% >30 years | Uganda | Periurban | Unclear | Bi-weekly mobile outreach clinics, brothel based testing, and nocturnal mobile vans for HIV testing. If HIV+ referred for follow up care at the static or mobile clinics (including same day ART start). Follow up included phone calls, peer educators, and home assessments. |  |

ANC= antenatal care; ART= antiretroviral therapy; FGD= focus group discussion; FSW= female sex workers; IQR= interquartile range; MSM= men who have sex with men; PMTCTB+= Prevention of Mother-To-Child HIV Transmission Option B+; PoC= point-of-care

1. Amanyire G, Semitala FC, Namusobya J, et al. Effects of a multicomponent intervention to streamline initiation of antiretroviral therapy in Africa: a stepped-wedge cluster-randomised trial. *Lancet HIV* 2016; **3**(11): e539-e48.

2. Koenig SP, Dorvil N, Devieux JG, et al. Same-day HIV testing with initiation of antiretroviral therapy versus standard care for persons living with HIV: A randomized unblinded trial. *PLoS Med* 2017; **14**(7): e1002357.

3. Rosen S, Maskew M, Fox MP, et al. Initiating Antiretroviral Therapy for HIV at a Patient's First Clinic Visit: The RapIT Randomized Controlled Trial. *PLoS Med* 2016; **13**(5): e1002015.

4. Labhardt ND, Ringera I, Lejone TI, et al. Same day ART initiation versus clinic-based pre-ART assessment and counselling for individuals newly tested HIV-positive during community-based HIV testing in rural Lesotho - a randomized controlled trial (CASCADE trial). *BMC Public Health* 2016; **16**(1): 329.

5. Chan AK, Kanike E, Bedell R, et al. Same day HIV diagnosis and antiretroviral therapy initiation affects retention in Option B+ prevention of mother-to-child transmission services at antenatal care in Zomba District, Malawi. *J Int AIDS Soc* 2016; **19**(1): 20672.

6. Hoenigl M, Chaillon A, Moore DJ, et al. Rapid HIV Viral Load Suppression in those Initiating Antiretroviral Therapy at First Visit after HIV Diagnosis. *Sci Rep* 2016; **6**: 32947.

7. Kerschberger B, Mazibuko S, Zabsonre I, et al. Outcomes of Patients Initiating ART under the WHO Test & Treat Approach. 21st International AIDS Conference, Durban. Abstract TUPEB060.

8. Langwenya N, Phillips T, Zerbe A, et al. Immediate initiation of antiretroviral therapy in PMTCT programmes is not associated with non-adherence during pregnancy: a cohort study. 8th IAS Conference on HIV Pathogenesis, Treatment, and Prevention. Vancouver, 19-22 July 2015. Abstract WEPED866.

9. Mitiku I, Arefayne M, Mesfin Y, Gizaw M. Factors associated with loss to follow-up among women in Option B+ PMTCT programme in northeast Ethiopia: a retrospective cohort study. *J Int AIDS Soc* 2016; **19**(1): 20662.

10. Pilcher CD, Ospina-Norvell C, Dasgupta A, et al. The Effect of Same-Day Observed Initiation of Antiretroviral Therapy on HIV Viral Load and Treatment Outcomes in a US Public Health Setting. *J Acquir Immune Defic Syndr* 2017; **74**(1): 44-51.

11. Wu Z, Zhao Y, Ge X, et al. Simplified HIV Testing and Treatment in China: Analysis of Mortality Rates Before and After a Structural Intervention. *PLoS Med* 2015; **12**(9): e1001874.

12. Black S, Zulliger R, Myer L, et al. Safety, feasibility and efficacy of a rapid ART initiation in pregnancy pilot programme in Cape Town, South Africa. *S Afr Med J* 2013; **103**(8): 557-62.

13. Girometti N, Nwokolo N, McOwan A, Whitlock G. Outcomes of acutely HIV-1-infected individuals following rapid antiretroviral therapy initiation. *Antivir Ther* 2016.

14. De Souza MS, Phanuphak N, Pinyakorn S, et al. Impact of nucleic acid testing relative to antigen/antibody combination immunoassay on the detection of acute HIV infection. *AIDS* 2015; **29**(7): 793-800.

15. Wilkinson L, Duvivier H, Patten Gea. Outcomes from the implementation of a counselling model supporting

rapid antiretroviral treatment initiation in a primary healthcare clinic in Khayelitsha, South Africa. S Afr J HIV Med. 16(1). 2015.

1. Amanyire G, Semitala FC, Namusobya J, et al. Effects of a multicomponent intervention to streamline initiation of antiretroviral therapy in Africa: a stepped-wedge cluster-randomised trial. *Lancet HIV* 2016; **3**(11): e539-e48.

2. Koenig SP, Dorvil N, Devieux JG, et al. Same-day HIV testing with initiation of antiretroviral therapy versus standard care for persons living with HIV: A randomized unblinded trial. *PLoS Med* 2017; **14**(7): e1002357.

3. Rosen S, Maskew M, Fox MP, et al. Initiating Antiretroviral Therapy for HIV at a Patient's First Clinic Visit: The RapIT Randomized Controlled Trial. *PLoS Med* 2016; **13**(5): e1002015.

4. Labhardt ND, Ringera I, Lejone TI, et al. Same day ART initiation versus clinic-based pre-ART assessment and counselling for individuals newly tested HIV-positive during community-based HIV testing in rural Lesotho - a randomized controlled trial (CASCADE trial). *BMC Public Health* 2016; **16**(1): 329.

5. Chan AK, Kanike E, Bedell R, et al. Same day HIV diagnosis and antiretroviral therapy initiation affects retention in Option B+ prevention of mother-to-child transmission services at antenatal care in Zomba District, Malawi. *J Int AIDS Soc* 2016; **19**(1): 20672.

6. Hoenigl M, Chaillon A, Moore DJ, et al. Rapid HIV Viral Load Suppression in those Initiating Antiretroviral Therapy at First Visit after HIV Diagnosis. *Sci Rep* 2016; **6**: 32947.

7. Kerschberger B, Mazibuko S, Zabsonre I, et al. Outcomes of Patients Initiating ART under the WHO Test & Treat Approach. 21st International AIDS Conference, Durban. Abstract TUPEB060.

8. Langwenya N, Phillips T, Zerbe A, et al. Immediate initiation of antiretroviral therapy in PMTCT programmes is not associated with non-adherence during pregnancy: a cohort study. 8th IAS Conference on HIV Pathogenesis, Treatment, and Prevention. Vancouver, 19-22 July 2015. Abstract WEPED866.

9. Mitiku I, Arefayne M, Mesfin Y, Gizaw M. Factors associated with loss to follow-up among women in Option B+ PMTCT programme in northeast Ethiopia: a retrospective cohort study. *J Int AIDS Soc* 2016; **19**(1): 20662.

10. Pilcher CD, Ospina-Norvell C, Dasgupta A, et al. The Effect of Same-Day Observed Initiation of Antiretroviral Therapy on HIV Viral Load and Treatment Outcomes in a US Public Health Setting. *J Acquir Immune Defic Syndr* 2017; **74**(1): 44-51.

11. Wu Z, Zhao Y, Ge X, et al. Simplified HIV Testing and Treatment in China: Analysis of Mortality Rates Before and After a Structural Intervention. *PLoS Med* 2015; **12**(9): e1001874.

12. Black S, Zulliger R, Myer L, et al. Safety, feasibility and efficacy of a rapid ART initiation in pregnancy pilot programme in Cape Town, South Africa. *S Afr Med J* 2013; **103**(8): 557-62.

13. Girometti N, Nwokolo N, McOwan A, Whitlock G. Outcomes of acutely HIV-1-infected individuals following rapid antiretroviral therapy initiation. *Antivir Ther* 2016.

14. De Souza MS, Phanuphak N, Pinyakorn S, et al. Impact of nucleic acid testing relative to antigen/antibody combination immunoassay on the detection of acute HIV infection. *AIDS* 2015; **29**(7): 793-800.

15. Wilkinson L, Duvivier H, Patten Gea. Outcomes from the implementation of a counselling model supporting

rapid antiretroviral treatment initiation in a primary healthcare clinic in Khayelitsha, South Africa. S Afr J HIV Med. 16(1). 2015.

**Risk of bias: randomized trials**

|  | **Allocation concealment described** | **Randomization described** | **Blinding of patients and providers** | **Outcome data complete** | **Non-selective outcome reporting** |
| --- | --- | --- | --- | --- | --- |
| Amanyire^1^ | NO | YES | NO | YES | YES |
| Koening^2^ | Yes | YES | NO | YES | YES |
| Rosen^3^ | YES | YES | NO | YES | YES |
| Labhardt^4^ | NO | YES | NO | NR | NR |

NR= not reported (study reported as conference abstract)

**Risk of bias: observational studies**

|  | **Prospective** | **Comparison group** | **Outcomes available for all patients** | **Follow up ≥ 6 months** |
| --- | --- | --- | --- | --- |
| Black^5^ | YES | NO | NO | NO |
| Wilkinson^6^ | YES | NO | YES | YES |
| Chan^7^ | NO | YES | NO | YES |
| Pilcher^8^ | YES | YES^#^ | NO | YES |
| Mitiku^9^ | NO | YES | YES | NO |
| Kerschberger^10##^ | YES | YES | YES | YES |
| Langwenya^11#^ | YES | YES | NO | NO |
| Hoenigl^12^ | NO | YES | NO | YES |
| Girometti^13^ | NO | NO | YES^###^ | YES |
| De Souza^14^ | YES | NO | YES^####^ | YES |
| Wu^15^ | YES | YES | YES | YES |

# The comparison group for pre- and post-intervention analysis across multiple time periods was a retrospective cohort

## Poster only. Additional data provided by the author

### Among those who started ART

#### 1 patient was LTFU

References

1. Amanyire G, Semitala FC, Namusobya J, et al. Effects of a multicomponent intervention to streamline initiation of antiretroviral therapy in Africa: a stepped-wedge cluster-randomised trial. *Lancet HIV* 2016; **3**(11): e539-e48.

2. Koenig SP, Dorvil N, Devieux JG, et al. Same-day HIV testing with initiation of antiretroviral therapy versus standard care for persons living with HIV: A randomized unblinded trial. *PLoS Med* 2017; **14**(7): e1002357.

3. Rosen S, Maskew M, Fox MP, et al. Initiating Antiretroviral Therapy for HIV at a Patient's First Clinic Visit: The RapIT Randomized Controlled Trial. *PLoS Med* 2016; **13**(5): e1002015.

4. Labhardt ND, Ringera I, Lejone TI, et al. Same day ART initiation versus clinic-based pre-ART assessment and counselling for individuals newly tested HIV-positive during community-based HIV testing in rural Lesotho - a randomized controlled trial (CASCADE trial). *BMC Public Health* 2016; **16**(1): 329.

5. Black S, Zulliger R, Myer L, et al. Safety, feasibility and efficacy of a rapid ART initiation in pregnancy pilot programme in Cape Town, South Africa. *S Afr Med J* 2013; **103**(8): 557-62.

6. Wilkinson L, Duvivier H, Patten Gea. Outcomes from the implementation of a counselling model supporting

rapid antiretroviral treatment initiation in a primary healthcare clinic in Khayelitsha, South Africa. S Afr J HIV Med. 16(1). 2015.

7. Chan AK, Kanike E, Bedell R, et al. Same day HIV diagnosis and antiretroviral therapy initiation affects retention in Option B+ prevention of mother-to-child transmission services at antenatal care in Zomba District, Malawi. *J Int AIDS Soc* 2016; **19**(1): 20672.

8. Pilcher CD, Ospina-Norvell C, Dasgupta A, et al. The Effect of Same-Day Observed Initiation of Antiretroviral Therapy on HIV Viral Load and Treatment Outcomes in a US Public Health Setting. *J Acquir Immune Defic Syndr* 2017; **74**(1): 44-51.

9. Mitiku I, Arefayne M, Mesfin Y, Gizaw M. Factors associated with loss to follow-up among women in Option B+ PMTCT programme in northeast Ethiopia: a retrospective cohort study. *J Int AIDS Soc* 2016; **19**(1): 20662.

10. Kerschberger B, Mazibuko S, Zabsonre I, et al. Outcomes of Patients Initiating ART under the WHO Test & Treat Approach. 21st International AIDS Conference, Durban. Abstract TUPEB060.

11. Langwenya N, Phillips T, Zerbe A, et al. Immediate initiation of antiretroviral therapy in PMTCT programmes is not associated with non-adherence during pregnancy: a cohort study. 8th IAS Conference on HIV Pathogenesis, Treatment, and Prevention. Vancouver, 19-22 July 2015. Abstract WEPED866.

12. Hoenigl M, Chaillon A, Moore DJ, et al. Rapid HIV Viral Load Suppression in those Initiating Antiretroviral Therapy at First Visit after HIV Diagnosis. *Sci Rep* 2016; **6**: 32947.

13. Girometti N, Nwokolo N, McOwan A, Whitlock G. Outcomes of acutely HIV-1-infected individuals following rapid antiretroviral therapy initiation. *Antivir Ther* 2016.

14. De Souza MS, Phanuphak N, Pinyakorn S, et al. Impact of nucleic acid testing relative to antigen/antibody combination immunoassay on the detection of acute HIV infection. *AIDS* 2015; **29**(7): 793-800.

15. Wu Z, Zhao Y, Ge X, et al. Simplified HIV Testing and Treatment in China: Analysis of Mortality Rates Before and After a Structural Intervention. *PLoS Med* 2015; **12**(9): e1001874.
